# Effects of Student Life on the Prevention of SARS-CoV-2 Spread at University

**DOI:** 10.1017/dmp.2020.494

**Published:** 2020-12-22

**Authors:** Moska Sial, Manavi Purohit, Matan Bone

**Affiliations:** Faculty of Biology, Medicine and Health, The University of Manchester, Manchester, United Kingdom

**Keywords:** disaster medicine, disaster planning, health education, infection control, medical student

## Abstract

The 2019 coronavirus disease (COVID-19) caused by severe acute respiratory syndrome coronavirus 2 (SARS-CoV-2) has been a pandemic in need of controlling. The disease has taken its toll on universities; as a consequence, universities must prepare their campuses in such a way that will reduce the spread of SARS-CoV-2 and COVID-19 and ensure the safety of their students. This is why it is necessary to critically assess the risks involved in reopening university campuses. This letter to the editor highlights the importance of the social side of student life on campus and how it might affect the precautions put in place to reduce SARS-CoV-2 transmission. Furthermore, this letter is proposing potential courses of action for universities to take during the pandemic for the forthcoming academic year. The ability of universities to contain the spread of the virus is limited, as they lack control over social interactions outside of campus. We discuss the multifaceted approach needed to educate students about off-campus transmission to prevent SARS-CoV-2 transmission.

The upcoming reopening of UK universities requires us to consider how students and staff can work together to halt a potential second spread of severe acute respiratory syndrome coronavirus-2 (SARS-CoV-2). This is crucial as there is a worrying possibility of renewed community transmission due to the relaxation of lockdown rules.^1^ This letter aims to highlight the importance of non-academic student life in the transmission of SARS-CoV-2 and urges university authorities to consider this issue before reopening university campuses.

As we, UK university students, return to campuses this fall, we read with interest the article, “SARS-CoV-2 Viral and Serological Testing When Colleges Reopen – Some Practical Considerations.”^2^ The article considers multiple methods that can be used in controlling SARS-CoV-2 transmission in university campuses, like weekly mass testing, self-isolation, and contact tracing.^2^ This approach is supported by Griffiths et al.^3^ While these steps are important, and the authors provided an assumption that the social aspect of student life is a determinant of SARS-CoV-2 prevention outcomes, they do not explicitly detail the magnitude of the problem nor directly provide practical solutions to address this predicament.

Students interact with people off-campus on a range of occasions; some of these involve social gatherings, cafeterias, extracurricular activities, and visiting friends and family. In the UK, as in some US universities, campuses are often located in large cities and so students’ interactions with locals are a significant factor. In many situations, it is impractical to expect students to adhere to precautionary measures, especially when guidelines are unclear. We believe that off-campus interactions can hinder the pursuit to contain SARS-CoV-2. Therefore, we recommend that guidelines concerning off-campus behavior among student populations be introduced.

It is critical for governments, universities, and students alike to consider off-campus infections as a significant public health emergency. Public health emergency is defined as situations “whose scale, timing, or unpredictability threatens to overwhelm routine capabilities,” and off-campus transmissions adhere to this definition.^4^ Officially defining this problem as an emergency would solidify its legitimacy as a major risk factor in SARS-CoV-2 transmission.

We recommend that this problem be addressed in a multifaceted manner. Primarily, universities must ensure that virus containment precautions are maintained outside of the campus. This can be achieved through education and advertising. Strategic campaigns can be used to improve the health literacy of the student population; using social media and holding interactive webinars are some of the approaches that require consideration. By conveying information this way, we seek to curb off-campus transmission of SARS-CoV-2.

Furthermore, we believe that universities must share responsibility for addressing this problem with student-led organizations, like the student unions and student representatives. The benefits of this approach are that policies are made jointly and not solely dictated by the universities. Concordance leads to better informed outcomes than authoritative compliance.^5^

The social side of student life has a substantial effect on SARS-CoV-2 transmission. Hence, careful consideration of the safety measures implemented at university campuses is needed to avoid potential SARS-CoV-2 outbreaks.
